# Proteogenomic identification of an immunogenic HLA class I neoantigen in mismatch repair–deficient colorectal cancer tissue

**DOI:** 10.1172/jci.insight.146356

**Published:** 2021-07-22

**Authors:** Tomomi Hirama, Serina Tokita, Munehide Nakatsugawa, Kenji Murata, Yasuhito Nannya, Kazuhiko Matsuo, Hidetoshi Inoko, Yoshihiko Hirohashi, Shinichi Hashimoto, Seishi Ogawa, Ichiro Takemasa, Noriyuki Sato, Fumitake Hata, Takayuki Kanaseki, Toshihiko Torigoe

**Affiliations:** 1 Department of Pathology, Sapporo Medical University, Sapporo, Japan.; 2 Sapporo Dohto Hospital, Sapporo, Japan.; 3 Department of Pathology, Tokyo Medical University Hachioji Medical Center, Tokyo, Japan.; 4 Department of Pathology and Tumor Biology, Graduate School of Medicine, Kyoto University, Kyoto, Japan.; 5 Sapporo Clinical Laboratory, Sapporo, Japan.; 6 GenoDive Pharma Inc., Atsugi, Japan.; 7 Department of Molecular Pathophysiology, Institute of Advanced Medicine, Wakayama Medical University, Wakayama, Japan.; 8 The World Premier International Research Center Initiative and Institute for the Advanced Study of Human Biology (WPI-ASHBi), Kyoto University, Kyoto, Japan.; 9 Department of Medicine, Centre for Haematology and Regenerative Medicine, Karolinska Institute, Stockholm, Sweden.; 10 Department of Surgery, Surgical Oncology and Science, Sapporo Medical University, Sapporo, Japan.

**Keywords:** Immunology, Cancer immunotherapy

## Abstract

Although CD8^+^ T cells recognize neoantigens that arise from somatic mutations in cancer, only a small fraction of nonsynonymous mutations give rise to clinically relevant neoantigens. In this study, HLA class I ligandomes of a panel of human colorectal cancer (CRC) and matched normal tissues were analyzed using mass spectrometry–based proteogenomic analysis. Neoantigen presentation was rare; however, the analysis detected a single neoantigen in a mismatch repair–deficient CRC (dMMR-CRC) tissue sample carrying 3967 nonsynonymous mutations, where abundant tumor-infiltrating lymphocytes (TILs) and inflamed gene expression status were observed in the tumor microenvironment (TME). Using the HLA class I ligandome data and gene expression profiles, a set of nonmutated tumor-associated antigen (TAA) candidates was concomitantly identified. Interestingly, CD8^+^ TILs predominantly recognized the detected neoantigen over the array of TAA candidates. Neoantigen-reactive CD8^+^ TILs showed PD-1 positivity and exhibited functional and specific responses. Moreover, T cell receptor (TCR) profiling identified the sequence of the neoantigen-reactive TCR clonotype and showed its expansion in the TME. Transduction of the sequenced TCR conferred neoantigen specificity and cytotoxicity to peripheral blood lymphocytes. The proteogenomic approach revealed the antigenic and reactive T cell landscape in dMMR-CRC, demonstrating the presence of an immunogenic neoantigen and its potential therapeutic applications.

## Introduction

Colorectal cancer (CRC) that is mismatch repair–deficient (dMMR) or microsatellite instability–high (MSI-H) accounts for approximately 15% of all CRCs (1). Mismatch repair deficiency is characterized by loss of function in MLH1, MSH2, PMS2, or MSH6 protein, leading to the accumulation of somatic mutations inside the cancer genome (2). Since dMMR-CRCs and other solid cancers are sensitive to immune checkpoint inhibitors (ICI), dMMR or MSI-H was approved by the FDA as the first tissue-agnostic biomarker across cancer types (3–5). Somatic nonsynonymous mutations in tumor cells give rise to mutated peptides, which may be presented by HLA and surveyed by host T cells. Because somatic mutations are unique to cancer, those mutated HLA ligands, or neoantigens, are not tolerated by hosts and often elicit T cell responses (6). Mutation-derived neoantigens are likely to be a major target of ICI-induced T cell responses (7, 8). Moreover, the adoptive transfer of tumor-infiltrating lymphocytes (TILs) containing neoantigen-specific T cell subsets has resulted in objective tumor regression in patients with epithelial cancers, including CRCs (9–11). Neoantigen vaccination allows for the activation of inherent host T cell responses in patients with melanoma or glioblastoma (12–14). All of these findings highlight the presence of clinically relevant neoantigens and their roles in patient T cell responses against tumor cells — in particular, against cells with high tumor mutation burden (TMB).

Neoantigens hold great promise as a more reliable predictive biomarker of response to ICI than TMB itself, or as therapeutic targets. However, the search for clinically relevant neoantigens within a vast mutation pool remains a daunting task. The majority of somatic mutations are passenger mutations unique to individuals. Therefore, neoantigens must be screened for individual patients. In addition, neoantigens must be presented by HLA at levels sufficient to elicit a host T cell response. Unfortunately, the specificity of current in silico neoantigen prediction solely based on the binding affinity between peptides and HLA molecules is not satisfactory because intracellular antigen processing is not fully understood and cannot be integrated into prediction (15).

Meanwhile, an approach using the biochemical isolation of HLA-bound peptides followed by tandem mass spectrometry (MS/MS) sequencing has allowed for the comprehensive and direct identification of antigens that are naturally processed and presented by cells of interest. The MS-based approach, which combines conventional proteomics with genomic data for searching neoantigens, is referred to as proteogenomics (16, 17). Seminal studies using mouse models have demonstrated the advantage of the proteogenomic approach in identifying neoantigens that are associated with tumor rejection in vivo (7, 18, 19). Likewise, neoantigen detection from human cell lines or patient-derived organoid models established in vitro has been demonstrated (20–22). However, clinical relevance of detected neoantigens remains elusive in most human cases; moreover, the successful detection from clinical tissues, which can serve as unbiased samples, to date has been limited to melanoma samples (23, 24). In the present study, we explored the HLA class I ligandomes of a panel of CRC tumor tissues and matched normal tissues using the proteogenomic approach. We show the feasibility of neoantigen identification directly from CRC tissue, demonstrating the presence of an immunogenic neoantigen in dMMR-CRC tissue, despite the rarity of neoantigen HLA presentation.

## Results

### Cytotoxic CD8^+^ T cell infiltration with inflamed gene expression signature in the TME of dMMR-CRC tissue.

**Figure 1:**
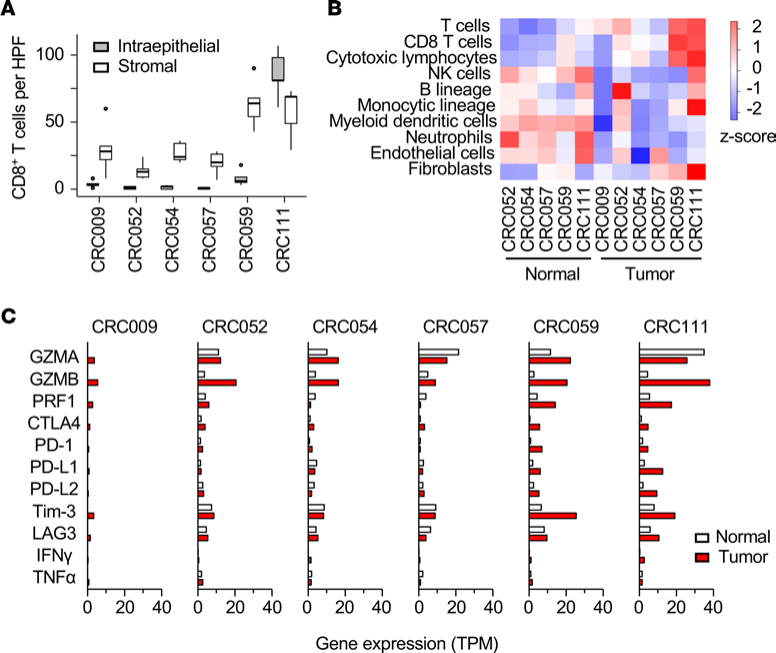
CD8^+^ T cell infiltration and gene expression signature in a panel of CRC and patient-matched normal tissues. (**A**) Numbers of CD8^+^ cells per HPF (an average of 10 HPFs) in a panel of CRC tumor tissues based on IHC in Supplemental Figure 1. CD8^+^ cells that resided in the tumor parenchyma (intraepithelial) and outside of the tumor parenchyma (stromal) were counted separately. Box-and-whisker plots represent the median (solid bars), interquartile range (boxes), and 1.5× interquartile range (vertical lines). Dots denote observations outside the range of adjacent values. (**B**) Estimated proportions of immune cells across the tumor and matched normal tissues calculated using MCP-counter with RNA-seq data. (**C**) Gene expression signature of cytotoxic granules, inflammatory cytokines, and checkpoint molecules between the tumor and matched normal tissues. The bar graphs represent the TPM values as determined via RNA-seq (*n* = 1).

Five surgically resected mismatch repair proficient (pMMR) and 1 dMMR primary CRC tissue were used as experimental samples. They were histologically diagnosed as adenocarcinoma with or without metastasis (Supplemental Figure 1; supplemental material available online with this article; https://doi.org/10.1172/jci.insight.146356DS1). A dMMR-CRC, CRC111, was characterized by the loss of MLH1 protein expression. In contrast to the other samples, the tumor region of the CRC111 tissue was accompanied by CD8^+^ T cell infiltration (Figure 1A). An increased number of CD8^+^ TILs inside the tumor parenchyma is a characteristic of inflamed-type cancer and is often observed in MSI-H CRCs (25–27). The tumor cells in the CRC111 tissue also showed an increased PD-L1 expression (Supplemental Figure 1). PD-L1 expression implies the presence of spontaneous T cell recognition, followed by the activation and production of IFN-γ on-site, which provides predictive values for the responses of patients with melanoma, non–small cell lung cancer, and bladder cancers to ICI monotherapy (28). Transcriptome analysis using RNA sequencing (RNA-seq) and the Microenvironment Cell Populations counter (MCP-counter) algorithm also suggested cytotoxic lymphocyte infiltration (29), most likely of CD8^+^ T cells, in the tumor region of the CRC111 tissue but not in the matched normal counterpart (Figure 1B). CD8^+^ T cell infiltration in CRC111 was accompanied by increases in cytotoxic granules (granzymes and perforin) and checkpoint gene expression (Figure 1C). Thus, our CRC tissue panel included an inflamed dMMR-CRC tissue with spontaneous T cell response, as well as noninflamed or moderately inflamed pMMR-CRC tissues.

### Conventional HLA class I ligandome analysis identifies the candidates of nonmutated TAAs.

**Figure 2:**
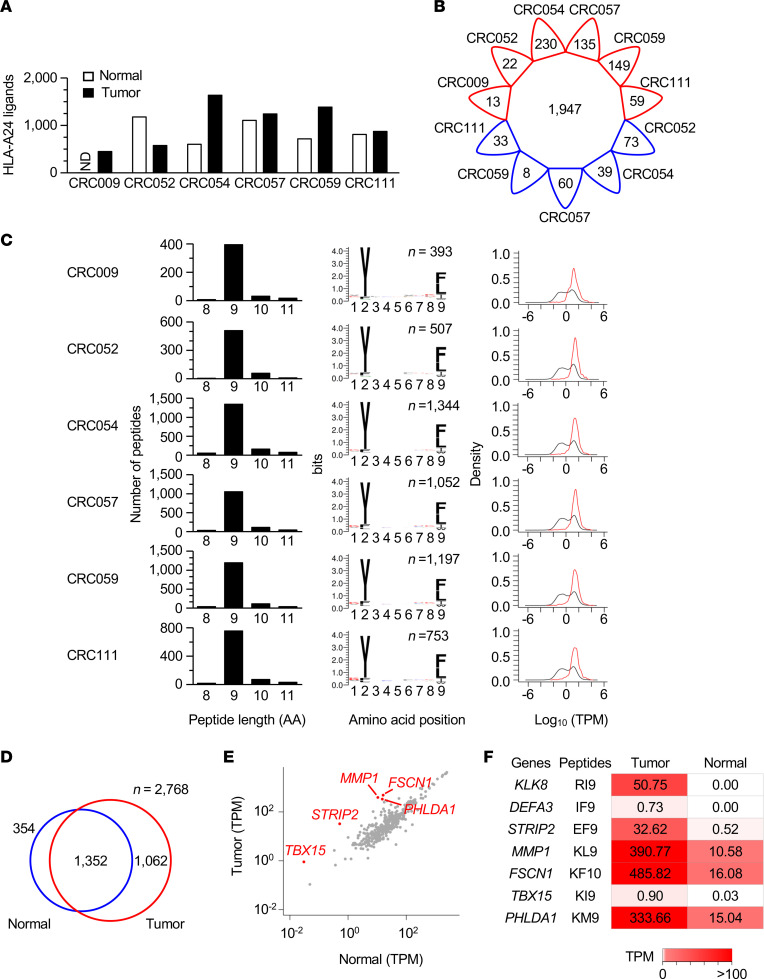
Characterization of natural HLA class I ligands and candidates for nonmutated TAAs found in CRC tissues. (**A**) Nonredundant numbers of nonmutated HLA-A24 ligands detected in a panel of CRC tumor and matched normal tissues by HLA ligandome analysis. Analysis was performed once per sample. (**B**) The number of HLA-A24 ligands shared between 2 or more samples is shown in the center of the diagram. The numbers of private HLA-A24 ligands unique to each sample are shown along the rim of the circle. Tumor and normal tissues are shown in red and blue, respectively. (**C**) Length distribution (left) and sequence logos of 9-mer peptides (middle) of HLA-A24 ligands. In the kernel density estimation of the transcripts expressed in the indicated samples (right), the black and red lines represent the whole transcriptome and transcripts encoding the HLA-A24 ligands, respectively. (**D**) Venn diagram showing the number of HLA-A24 ligands overlapping between tumor and normal tissues. (**E**) Differential gene expression between CRC111 tumor and matched normal tissues. Only the genes encoding HLA-A24 ligands that were detected in CRC111 tumor tissue, but not in normal tissues, are plotted. Genes with a more than 20-fold increase in tumors are shown in red. (**F**) Candidates of nonmutated TAAs and their source genes. The numbers in the heatmap indicate the TPM values of the genes in CRC111 tumor and normal tissues.

Conventional HLA ligandome analysis was performed without using mutation data to identify the HLA class I immunopeptidome of CRC tissues for canonical tumor-associated antigens (TAAs) irrelevant to somatic mutations. All of the patients enrolled in this study were HLA-A*24:02 positive (Supplemental Table 1). The HLA ligandome analysis via MS coupled with affinity purification of peptide–HLA-A24 complexes (pHLA-A24) revealed an average of 964 unique peptides per sample presented by CRC tumor or matched normal tissues (Supplemental Table 2 and Figure 2A). In total, 2768 nonredundant peptide sequences were identified, 70.3% of which were shared across 2 or more tumor or normal tissues, indicating a trend in peptide presentation (Figure 2B). Among the immunopeptidomes of the 6 tumor tissues, 9-mer peptides were found to be dominant in length, and HLA-A24 binding anchor motifs were conserved at P2 and PΩ (Figure 2C). The genes encoding the natural HLA ligands were biased toward those with high expression levels, suggesting a positive correlation between HLA peptide presentation and transcriptional abundance (30, 31).

By comparing tumor and normal tissue immunopeptidomes, 61.6% (1706 of 2768) of HLA ligands were shared or found in normal samples, leaving 1062 peptides as potential candidates for canonical TAAs (Figure 2D). Since spontaneous T cell response and the inflamed TME were observed in CRC111, we combined the peptidome data with gene expression profiles, comparing the transcriptional abundance of the 789 HLA ligands that were detected in the CRC111 tumor but not in any of the normal tissues (Figure 2E). Despite the fact that the gene expression levels were mostly comparable between CRC111 tumor and normal samples, some genes encoding the HLA ligands were found to increase over 20-fold in the tumor (Figure 2F). These HLA-A24 ligands were possibly overexpressed in tumor samples and were therefore chosen as nonmutated TAA candidates of CRC111, which comprised peptides encoded by the *KLK8*, *DEFA3*, *STRIP2*, *MMP1*, *FSCN1*, *TBX15*, and *PHLDA1* genes (Supplemental Table 3). These peptides were shared across 2 or more CRC tumor tissues in our panel. The peptides except for KL9 have been reported as natural HLA class I ligands identified using MS across cancer types (32–34).

### Neoantigen identification using a proteogenomic approach.

**Figure 3:**
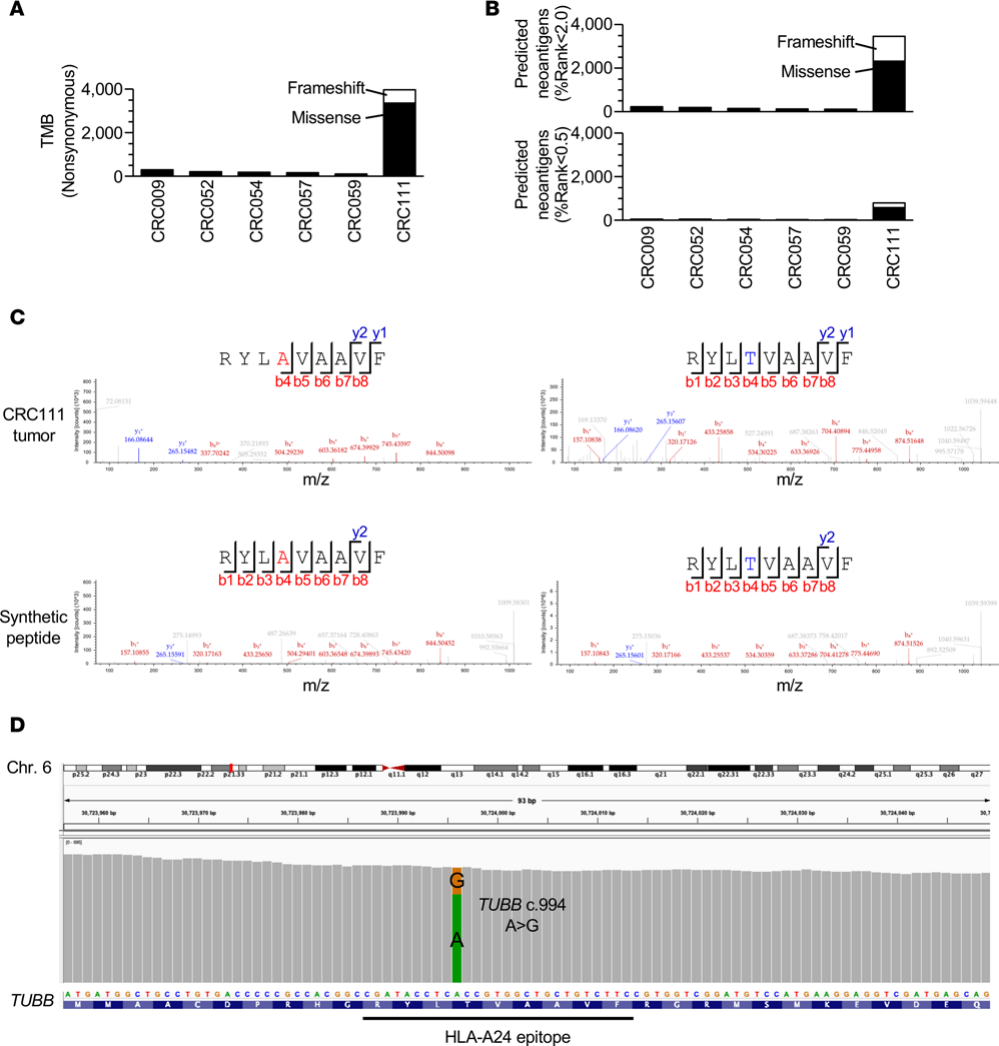
Proteogenomic identification of a neoantigen directly from dMMR-CRC tissue. (**A**) Numbers of nonsynonymous (missense and frameshift) somatic mutations in CRC tumor tissues with pMMR (CRC009, -052, -054, -057, and -059) and dMMR (CRC111). (**B**) Numbers of neoantigens predicted in silico using WES and RNA-seq data under the condition of NetMHCpan-4.1 %rank < 2.0 or < 0.5, the TPM of the source genes > 0 and the peptide length of 8–11 amino acids. (**C**) Neoantigen and its WT counterpart sequences identified in CRC111 tumor tissue using a proteogenomic approach at an FDR of 0.01. MS/MS spectra and the corresponding b- and y-fragment ions of both endogenous and synthetic peptides are shown. (**D**) Coverage track of the *TUBB* gene mutation in CRC111 tumor tissue visualized using the Integrative Genomics Viewer (IGV) (http://software.broadinstitute.org/software/igv/).

Mutation calling by whole-exome sequencing (WES) was performed to obtain somatic mutation data unique to each sample. In contrast to 5 CRC tissues with pMMR, CRC111 exhibited a considerably high TMB, containing 3367 and 600 missense and frameshift mutations, respectively (Figure 3A). First, mutation-derived neoantigens were predicted in silico solely using the WES and RNA-seq data. Due to the high TMB, prediction considering NetMHC scores (%rank score < 2.0), gene expression profiles (transcripts per million [TPM] > 0), and peptide lengths (8–11 amino acids) yielded 3465 HLA-A24 neoantigen candidates in the CRC111 tumor sample (Figure 3B). This computational approach predicted that 31.0% (1230 of 3967) of the mutations encoded at least 1 HLA-A24–presented neoantigen. Next, to further focus on naturally presented neoantigens, all the expressed transcripts harboring missense and frameshift mutations were virtually translated per sample and used to build personalized reference databases in the search for neoantigens from MS-based HLA ligandome data (Supplemental Figure 2). Although neoantigens were not detected in pMMR-CRC tissues, the proteogenomic approach identified a 9-mer neoantigen, RAF9, in the HLA-A24 ligandome of CRC111 (Figure 3C). At a stringent FDR of 0.01, RAF9 was the only neoantigen detected in the CRC111 tissue. RAF9 arose from a missense mutation (c.994 A>G) of the *TUBB* gene, which led to a single amino acid substitution (p.T332A) at peptide position 4 (Figure 3D). The variant allele frequency based of the mutation was 94 of 434 and 0 of 544 reads in CRC111 tumor and matched normal tissues, respectively. The *TUBB* c.994 A>G mutation was not registered in the COSMIC (https://cancer.sanger.ac.uk/cosmic) nor in the TCGA (https://portal.gdc.cancer.gov) databases, suggesting that it was a passenger event unique to CRC111. *TUBB* transcripts were abundantly expressed across a variety of normal organs, as well as in our CRC panel with a mean TPM of 474.5 (Supplemental Figure 3). In accordance with the positive correlation between HLA presentation and gene expression, the WT counterpart of RAF9 was also detected across CRC tumor and normal tissue samples (Table 1). Thus, we concluded that the proteogenomic approach directly identified a missense-mutation–derived neoantigen, RAF9, which was naturally processed and presented by HLA-A24 of tumor cells in the dMMR-CRC. In our analysis, neoantigens derived from frameshift mutations, which often increase in MSI-CRCs and correlate with CD8^+^ T cell infiltration (35), were not detected.

### Tumor-infiltrating CD8^+^ T cells predominantly recognize an identified neoantigen over nonmutated TAA candidates.

**Figure 4:**
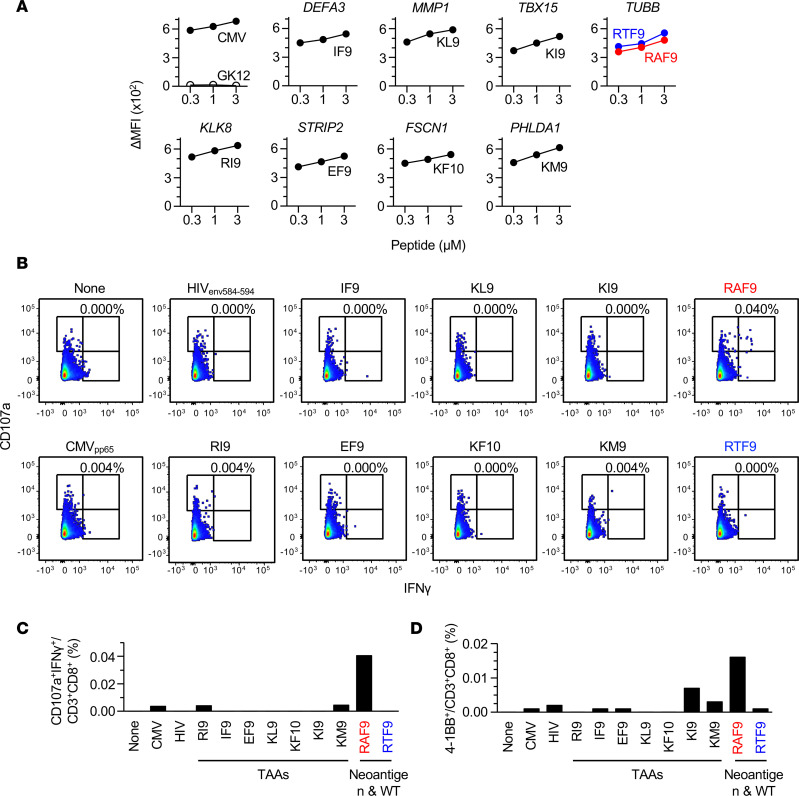
CRC111 TILs predominantly recognize the RAF9 neoantigen over nonmutated TAA candidates. (**A**) Peptide-HLA-A24 stabilization assay using T2-A24 cells pulsed with an indicated concentration range of synthetic peptides. Cells were stained with anti–HLA-A24, and the change in mean fluorescence intensity (ΔMFI) was calculated as the experimental MFI minus background MFI without a peptide pulse. A neoantigen and its WT counterpart are shown in red and blue, respectively. CMV_pp65_ and GK12 serve as controls for known HLA-A24 binding and nonbinding peptides, respectively. Data are representative of 2 independent experiments. (**B**) Intracellular IFN-γ production of CRC111 TILs in response to T2-A24 cells pulsed with 1 μM of the indicated peptides. Neoantigen, red; WT, blue. The results for CD3^+^CD8^+^ cells, and more than 20,000 CD3^+^CD8^+^ cells, were collected in each panel. Numbers in the upper right quadrant indicate the proportion of IFN-γ^+^CD107a^+^ cells in CD3^+^CD8^+^ cells. (**C**) Summary of IFN-γ production by TILs in **B**. Data in **B** and **C** are representatives of 2 independent experiments. (**D**) Summary of 4-1BB surface expression on CRC111 TILs in response to T2-A24 cells pulsed with 1 μM of indicated peptides, measured using flow cytometry. The *y* axis indicates the proportion of 4-1BB^+^ (CD137^+^) cells in CD3^+^ CD8^+^ cells. Data are representative of 2 independent experiments.

The presence of patient immune surveillance against tumor cells may lead to the accumulation of CD8^+^ T cells in the TME. Neoantigens are a possible target of CD8^+^ TILs in patients with gastrointestinal cancers (36, 37). To clarify the antigen specificity in the inflamed TME of the CRC111 tissue, we expanded TILs and assessed their recognition against the identified candidates. Every candidate antigen successfully formed stable pHLA-A24 on the surface of T2-A24 cells (Figure 4A). Comparable pHLA-A24 stability between the neoantigen (RAF9) and WT peptide (RTF9) indicated that the mutation at position 4 did not affect HLA binding — in particular, RAF9 was a non–anchor-type neoantigen. Interestingly, the assessment based on intracellular IFN-γ production, as well as CD107a expression, demonstrated the predominant CRC111-CD8^+^ TIL recognition against the RAF9 neoantigen (Figure 4, B and C). At the detection threshold of the assay, neither canonical TAA candidates, WT RTF9, nor an HIV antigen as a negative control were recognized by the TILs. Likewise, the measurement of cell-surface 4-1BB expression as an activation marker showed the same trend (Figure 4D). The upregulation of 4-1BB secondary to RAF9 was observed in KI9; however, the TILs did not produce IFN-γ in response to the KI9 peptide. Thus, the CD8^+^ TILs predominantly responded to RAF9 presented by T2-A24 cells, while they ignored or barely responded to the other antigens. The identified RAF9 neoantigen was a recognizable target of CRC111-CD8^+^ TILs, indicating the presence of patient immune surveillance against it. Despite the fact that our experimental setting was limited to the HLA-A24 ligandome, the results suggested that the antigen specificity of CRC111 patient TILs was likely biased toward the neoantigen over canonical TAAs.

### Cytotoxic function and phenotype of neoantigen-reactive CD8^+^ TILs.

**Figure 5:**
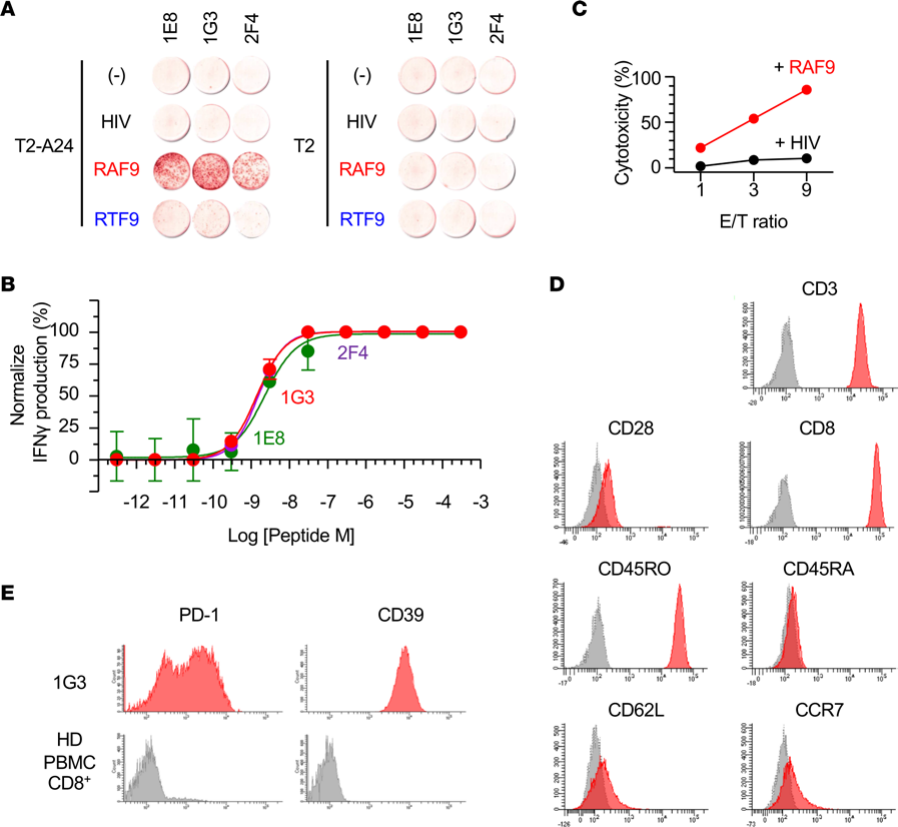
Functions and phenotypes of the RAF9-reactive CD8^+^ TIL clones. (**A**) IFN-γ ELISpot assay of RAF9-reactive CD8^+^ T cell clones derived from CRC111 TILs (1E8, 1G3, and 2F4) in response to T2-A24 or T2 cells pulsed with 100 nM of the indicated peptides. Data are representative of 4 independent experiments. (**B**) Functional avidity of 1E8, 1G3, and 2F4 measured by IFN-γ-ELISpot assay against T2-A24 pulsed with a range of indicated concentrations of RAF9 peptide. The number of positive spots was normalized (%). Data represent means ± SEM (*n* = 3). (**C**) LDH-release cytotoxicity of 1G3 against T2-A24 cells pulsed with RAF9 or a control peptide at the indicated effector/target (E/T) ratio. The *y* axis indicates LDH release (%) by target cells. Data are representative of 3 independent experiments. (**D**) Flow cytometry of 1G3 stained with the indicated antibodies (red) or PBS (gray). (**E**) Flow cytometry of 1G3 (red) and HD-derived CD8^+^ PBMCs. Data are representative of 2 independent experiments.

Next, we investigated the functional properties of the neoantigen-reactive TILs derived from CRC111. To this end, 3 CD8^+^ TIL clones recognizing RAF9 were established using single-cell sorting of 4-1BB^+^ subsets followed by in vitro expansion. In the IFN-γ–ELISpot assay, all 3 clones produced IFN-γ in response to T2-A24 cells pulsed with RAF9 peptide, but not to T2 cells that lacked HLA-A24 expression (Figure 5A). HLA class I restriction of the response was validated through a blocking assay using specific antibodies (Supplemental Figure 4). In addition, the responses were specific to RAF9, with limited cross-reactivity to RTF9. We also measured the functional avidities of 3 IFN-γ–producing RAF9-reactive clones. The clones 1E8, 1G3, and 2F4 demonstrated EC_50_ values of 2.27 nM, 1.40 nM, and 1.54 nM, respectively, which were consistent with the neoantigen-specific high-functional avidity often observed in the TIL population (Figure 5B) (38). Moreover, the lactate dehydrogenase–mediated (LDH-mediated) cytotoxicity assay showed the cytotoxicity of 1G3, in which 85.5% of T2-A24 cells pulsed with RAF9 peptide were lysed at the effector/target (E/T) ratio of 9:1 (Figure 5C).

The RAF9-reactive TIL clones carried an antigen-experienced T cell phenotype resembling effector memory CD8^+^ T cells (CD3^+^, CD8^+^, CD28^+^, CD45RO^+^, CD45RA^–^, CD62L^–^, and CCR7^–^) (Figure 5D). The presence of TILs with a CD3^+^CD45RO^+^ memory phenotype may play a role in preventing recurrence or metastasis in CRCs (39). Although TILs often comprise a heterogeneous mixture of tumor-specific and many irrelevant bystander T cells, tumor-reactive CD8^+^ TILs may be characterized by the upregulation of programmed cell death 1 (PD-1) and CD39 expression (40, 41). Consistent with the previous findings, RAF9-reactive clones showed PD-1 and CD39 expression on the cell surface (Figure 5E). These results support the presence of functional and specific TIL response against the RAF9 neoantigen in the CRC111 patient.

### RAF9 is an immunogenic neoantigen leading to the expansion of reactive CD8^+^ TILs in the TME.

**Figure 6:**
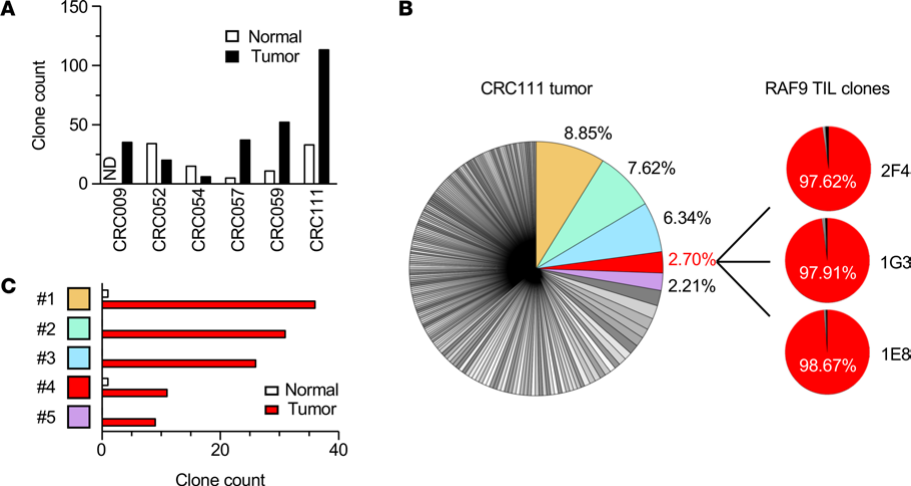
TCR repertoire analysis of CRC tissues and the sequence of the RAF9-reactive CD8^+^ TCR. (**A**) Clone counts of frequently observed TCRβ clonotypes, which accounted for more than 2% of all the TCRβ clonotypes found in the indicated CRC tumor or normal tissue samples. ND, no data. (**B**) Pie charts showing the frequency of TCRβ clonotypes found in CRC111 tumor tissue (left), and the clonotypes of 3 RAF9-reactive TIL clones (right). The abundant clonotypes with more than 2% of frequency were colored. (**C**) Clone counts of the top 5 TCRβ clonotypes frequently observed in CRC111 tumor tissue are shown in comparison with those in CRC111 normal tissue.

In a seminal study of patients with advanced melanoma, a more clonal TCR repertoire was observed in pretreatment tumor samples of responders, suggesting the requirement of preexisting antitumor responses for ICI-mediated tumor regression (42). In addition, melanoma cells can be eliminated by a limited number of patient neoantigen-reactive T cell clonotypes (24). Therefore, only a few tumor antigens and specific T cells could mediate spontaneous antitumor responses and lead to therapeutic effects. To assess TIL diversity in our CRC panel, the frequency of TCRβ clonotypes was estimated using RNA-seq data and the MiXCR algorithm with complementarity-determining region 3 (CDR3) as well as V, D, J, and C gene sequences (43). Given an extremely diverse repertoire of TCRαβ in normal PBMCs, we focused on TCRβ clonotypes accounting for over 2% of the entire repertoire (44, 45). These expanded TCRβ clonotypes were mainly observed in tumor regions rather than matched normal tissues — in particular, in the tumor sample of CRC111, suggesting the presence of T cell surveillance against tumor cells (Figure 6A). None of the expanded TCRβ clonotypes were shared across individuals. The TCR repertoire of CRC111 TME contained at least 5 expanded TCRβ clonotypes (Figure 6B). All 5 clonotypes were observed mainly in tumors but not in patient-matched normal tissues, suggesting the tumor specificity of these TILs (Figure 6C). We found that one of the expanded clonotypes was fully matched to that of RAF9-reactive CD8^+^ TIL clones, in which all 3 established T cell clones shared the identical TCRβ clonotype (Figure 6B and Table 2). The RAF9-reactive CD8^+^ T cell clones accounted for approximately 2.7% of the TCR repertoire of the CRC111 tumor tissue. These results demonstrate that RAF9 was an immunogenic neoantigen, which led to the clonal expansion of RAF9-reactive CD8^+^ T cell clones in the TME of the CRC111 patient. The antigen specificity and HLA restriction of the other 4 expanded CRC111-clonotypes remained unknown.

### TCR transduction conferred neoantigen specificity to peripheral blood lymphocytes.

**Figure 7:**
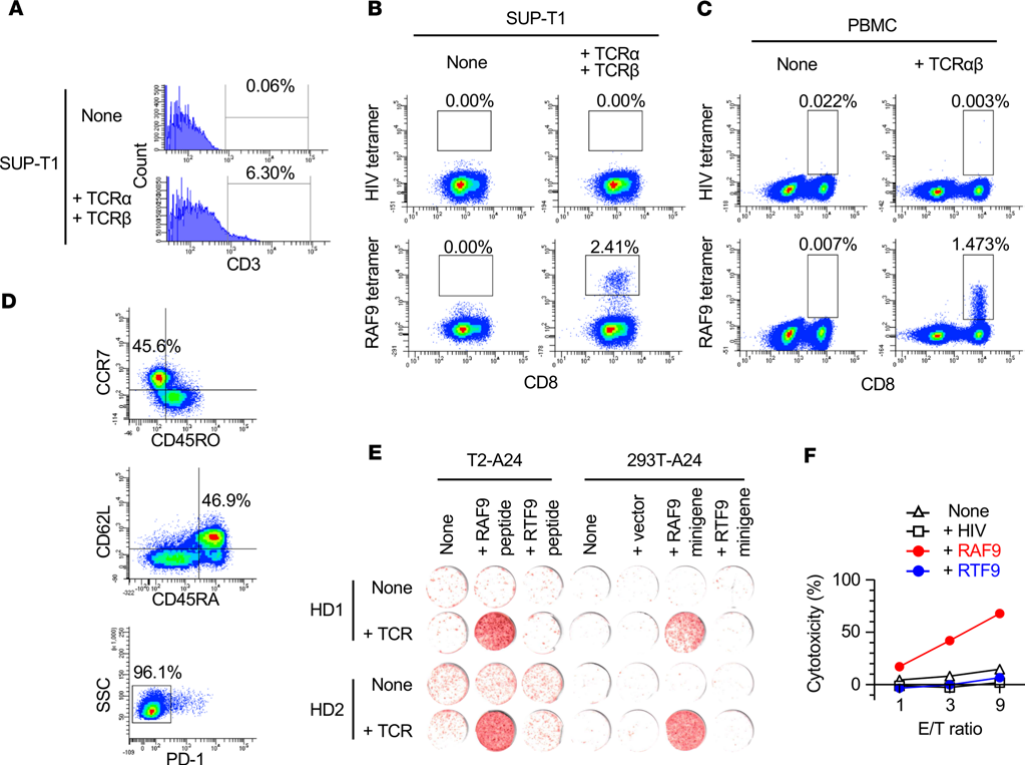
Isolation of RAF9-reactive TCR and transduction to peripheral blood lymphocytes. (**A** and **B**) Flow cytometry of SUP-T1 cells transduced with retroviral vectors encoding the TCRα and -β sequences isolated from a RAF9-reactive TIL clone (1E8). The numbers (%) represent the frequency of CD3^+^ cells (**A**) or RAF9–HLA-A24 tetramer^+^ cells in CD8^+^ cells (**B**). (**C**) Flow cytometry of healthy donor 1–derived (HD1-derived) PBMCs transduced with a retroviral vector encoding concatenated RAF9-reactive TCRα and -β sequences. The numbers (%) represent the frequency of RAF9–HLA-A24 tetramer^+^ cells in CD8^+^ cells. (**D**) Flow cytometry of the CD8 and RAF9 tetramer–double-positive fraction of HD1-PBMCs transduced with RAF9-TCRαβ. The numbers (%) represent the frequency of cells in the indicated fraction in the whole CD8 and tetramer double-positive cells. (**E**) IFN-γ ELISpot assay of the transduced PBMCs in response to T2-A24 cells pulsed with 100 nM of the indicated peptides (left) or 293T-A24 cells expressing minigenes encoding RAF9 or RTF9 peptide (right). The experiments were independently performed using PBMCs from 2 individuals (HD1 and HD2). (**F**) LDH-release cytotoxicity of the transduced PBMCs against T2-A24 cells pulsed with the indicated peptides at the indicated effector/target (E/T) ratio. The *y* axis represents LDH release (%) by the target cells. Data are representative of 3 independent experiments.

TME facilitates tumor escape from T cell surveillance through tolerance or inhibiting existing responses, so host neoantigen-reactive TILs often become dysfunctional. In contrast, peripheral blood lymphocytes (PBLs) include less-differentiated naive T cell subsets that have high proliferative potential. Therefore, PBLs transduced with neoantigen-reactive TCRs may serve as an alternative source of effector cells in adoptive cell transfer targeting neoantigens (46, 47). To assess the feasibility of outsourcing RAF9-reactive T cells, we first validated the antigen specificity of the identified TCR by transferring their sequences into irrelevant lymphocytes. SUP-T1 cells were transduced with 2 pMX retroviral vectors encoding the TCRα and TCRβ sequences found in RAF9-reactive clones. The increase in CD3 expression suggested that approximately 6% of the cells expressed paired TCRαβ on their surfaces (Figure 7A). The transduced SUP-T1 was positive for the RAF9–HLA-A24 tetramer, demonstrating the RAF9 recognition by the TCR (Figure 7B). Next, the TCRα and -β sequences were concatenated and cloned into a pMX retroviral vector and transduced into healthy donor–derived (HD-derived) PBMCs, in which 1.5% of the CD8^+^ cells were also positive for the RAF9–HLA-A24 tetramer (Figure 7C). Most of the tetramer^+^CD8^+^ cells exhibited a naive phenotype (CD45RA^+^, CD45RO^–^, CD62L^+^, CCR7^+^) and were negative for PD-1 (Figure 7D). Finally, the ELISpot assay showed that the TCR-transduced PBLs produced IFN-γ in response to T2-A24 cells pulsed with RAF9 but not RTF9 synthetic peptide. The transduced cells also responded to the 293T-A24 cells endogenously expressing the RAF9 minigene, producing IFN-γ (Figure 7E). Antigen specificity and function were validated using PBMCs derived from 2 different individuals. Moreover, the RAF9 tetramer^+^CD8^+^ fraction of the transduced PBMCs were enriched using a cell sorter (Supplemental Figure 5). The enriched PBMCs exhibited cytotoxicity specific to RAF9, in which 67.7% of RAF9-pulsed T2-A24 cells were lysed at an E/T ratio of 9:1 (Figure 7F). Thus, the identified TCR pair was RAF9 specific, and that TCR transfer conferred neoantigen-specific cytotoxicity to the naive PBLs. These results highlight the potential therapeutic applications of neoantigens identified through the proteogenomic approach and clonally expanded neoantigen-reactive TCRs.

## Discussion

We found an immunogenic neoantigen that induced a spontaneous patient CD8^+^ T cell response and expansion in the TME of a surgically resected dMMR-CRC tissue. Importantly, such an immunogenic neoantigen could be identified directly from the native CRC tissue sample without relying on in vitro tumor cell culture or reactive T cell expansion. A major advantage of a proteogenomic approach using MS compared with in silico prediction relying solely on genomic data is the direct capture of HLA-presented peptides in a comprehensive manner, which significantly reduces the number of corresponding candidate mutations (18). A peptide repertoire displayed by HLA class I is an outcome of an endogenous peptide competition; this is elaborately controlled by antigen processing, which comprises multiple steps across intracellular compartments (48). The expression of the antigen processing machinery is heterogeneous across cancer types — or even within the same tumor mass. For instance, the loss of the transporter associated with antigen processing or tapasin is often observed in cancers, which leads to alterations of peptide repertoires and T cell responses against tumor cells (49–51). Therefore, the direct identification of naturally presented peptides from samples benefits in detecting clinically relevant antigens over prediction from source genes, which must take the complicated antigen processing into account.

In the case of CRC111 carrying 3967 nonsynonymous missense and frameshift mutations, the naturally presented neoantigen accounted for only 1 of 877 detected HLA-A24 ligands. Although the precise size of HLA class I ligandomes is not fully understood, the frequency of neoantigen HLA presentation seems to be rare even in tumors with dMMR. Meanwhile, we cannot not rule out the possibility of overlooked neoantigens in our analysis due to limitations in sensitivity. Tissue samples contain not only tumor cells, but also normal stromal cells, which may dilute the proportion of tumor cells within the sample. During MS analysis, ionized peptides that are abundant in a sample are preferentially detected. A small number of peptides may be ignored compared with the next-generation sequencing, in which nucleotides can be amplified. Peptides with insufficient MHC binding affinity could also be lost during the affinity purification process. In fact, NetMHC combined with gene expression data predicted 3465 candidates as HLA-A24–presenting neoantigens in the CRC111 tumor. Again, our results do not necessarily deny the possibility of the HLA presentation of the in silico–predicted neoantigen candidates. Nevertheless, considering the limited number of expanded TCR clonotypes in the TME (e.g., only 5 TCRβ clonotypes exceeded 2% of the whole repertoire), an overwhelming majority of the 3465 predicted sequences should be irrelevant to the spontaneous patient responses leading to the T cell expansion. It is plausible that, even if presented by HLA molecules on cell surfaces, not every neoantigen becomes immunogenic. The intrinsic determinants of immunogenicity remain unclear; however, the presence of immunogenic neoantigens likely leads to ICI-mediated T cell responses in dMMR-CRCs. As previously observed in patients with melanoma, our data support the notion that a limited set of neoantigens serves as the target of patient immune surveillance even in dMMR-CRCs with high TMB (24).

Patient immune surveillance often edits and eliminates immunogenic neoantigens throughout clinical courses (52, 53). Our data show that RAF9 was not eliminated by the patient’s initial response and was consequently detected in the established cancer tissue (pT4N0M0). The exhaustion of RAF9-reactive CD8^+^ T cells characterized by upregulated PD-1 and CD39 expression partly accounts for the escape from immune surveillance. Given their intact HLA presentation, most MS-detectable immunogenic neoantigens may be accompanied by the exhaustion or dysfunction of reactive T cells in the TME. In contrast, immunogenic neoantigens are likely undetectable from tumor samples that have a loss of neoantigen gene expression or loss of HLA presentation (54–56). Hence, vaccination or adoptive T cell transfer, which induces or provides functional T cells, holds great promise as a therapeutic strategy targeting MS-identified neoantigens. Although neoantigen vaccination in patients with melanoma successfully broadens T cell responses, it remains a challenge to precisely predict responsible epitopes prior to vaccination (12–14). Therefore, the efficient selection of immunogenic neoantigens is of clinical importance. It has been shown that vaccination with MS-identified neoantigens, rather than those predicted using an MHC binding affinity algorithm, effectively mediated tumor rejection in a mouse tumor model, suggesting the reliability of MS-identified neoantigens as a source of therapeutic targets (19). Moreover, we demonstrated the feasibility of transferring a TCR recognizing an MS-identified neoantigen. This strategy allows the outsourcing of tumor-reactive T cells from PBLs, which generally contain less-differentiated naive T cells, and it may provide opportunities for the future development of T cell–based therapies.

In the present study, the detected neoantigen-dominated TIL responses over nonmutated TAAs, implying the neoantigen-oriented antigenic and reactive T cell landscape of the dMMR-CRC tissue. However, we must mention that the antigen specificity of 4 of 5 TCRβ clonotypes expanded in the TME of CRC111 remains unknown (Figure 6B). Considering their biased clonotype counts enriched in tumor tissues, these TCRs likely recognize tumor cells but not their normal counterparts (Figure 6C). Although the sensitivity issue must be considered, RAF9 was the only neoantigen captured from CRC111 tumor tissue. It is possible that these expanded T cells still recognize neoantigens, which were presented by HLA other than HLA-A24 and were, therefore, not detected in our analysis. Another interpretation follows from the presence of immunogenic antigens that are irrelevant to somatic mutations. Considering that PD-1^+^CD8^+^ T cell infiltration in the TME was correlated with ICI-mediated pathological responses even in pMMR-CRCs, patient immune surveillance related to therapeutic effects is not frequent yet present against TMB-low CRC cells (57). The source of tumor antigens is indeed diverse; for instance, cryptic translation products, which were not analyzed in this study, may be presented by HLA class I molecules and serve as targets for host T cell surveillance against tumors (58, 59). These remaining issues should be investigated in future studies.

In summary, the antigenic landscape and antigen specificity of CD8^+^ TILs in CRC tissues were explored. We found an immunogenic neoantigen in a dMMR-CRC tissue, which predominantly elicited a CD8^+^ TIL response and clonal expansion in the TME. Our results show that such clinically relevant neoantigens can be captured directly from native CRC tissue. The direct identification of clinically relevant neoantigens from CRC tissue samples, and possibly from other epithelial cancer tissues, using a proteogenomic approach could contribute to the design and development of a predictive biomarker for ICI responses, as well as clinical interventions, such as vaccines or adoptive cell transfer.

## Methods

### Patient material.

All CRC patients included in this study were histologically diagnosed with adenocarcinoma. Patient material was immediately sampled after surgery and analyzed at Sapporo Medical University. Approximately 0.5–2.0 g each of tumor and nontumor regions were selected with careful removal of extra connective tissues by pathologists and used for TIL expansion. The remaining samples were immediately snap frozen and cryopreserved. DNA samples prepared using the Allprep DNA/RNA/Protein Kit (Qiagen) from patient PBMCs were used for HLA genotyping. All patients enrolled in this study were confirmed to be HLA-A*24:02 positive.

### Cell lines.

The T2 cell line was purchased from ATCC (CRL-1992), while T2-A24 cell line (TAP-deficient T2 stably expressing HLA-A*24:02) was gifted by K. Kuzushima (Aichi Cancer Center Research Institute, Nagoya, Japan). The 293T cell line was purchased from ATCC, and 293T-A24 stably expresses HLA-A*24:02. Unless mentioned otherwise, the cells were cultured in the complete RPMI 1640 (Nacalai tesque) or DMEM (Nacalai tesque) supplemented with 10% FBS, 1% penicillin/streptomycin (Thermo Fisher Scientific), 1% GlutaMAX (Thermo Fisher Scientific), 10 mM HEPES (Thermo Fisher Scientific), 1 mM sodium pyruvate (Thermo Fisher Scientific), and 55 µM 2-mercaptoethanol (Thermo Fisher Scientific) in 5% CO_2_ incubators at 37°C.

### TIL.

After carefully removing the surrounding connective tissues, primary CRC tissues were manually minced and cultured in complete AIM-V medium (Thermo Fisher Scientific) supplemented with 6000 U/mL IL-2 (Peprotech) and 2.5 μg/mL amphotericin B (Thermo Fisher Scientific) for 4–6 weeks. The complete AIM-V medium contained 10% human AB serum (Biowest), 1% penicillin/streptomycin, 1% GlutaMAX (Thermo Fisher Scientific), 10 mM HEPES, 1 mM sodium pyruvate, and 55 μM 2-mercaptoethanol. TILs were further expanded using the rapid-expansion protocol as previously described (60). Briefly, the cells were cultured in the complete AIM-V supplemented with 6000 U/mL IL-2, 30 ng/mL anti-CD3 (OKT3, BioLegend 317302), and 2.5 μg/mL amphotericin B along with irradiated (100 Gy) HD-derived PBMCs for an additional 2 weeks. Expanded TILs were cryopreserved until use.

### IHC.

Formalin-fixed paraffin-embedded cancer tissues were mounted and stained with H&E, anti–pan HLA class I (EMR8-5, Hokudo), anti-CD8 (C8/144B, DAKO), anti–PD-L1 (E1L3N, Cell Signaling Technology 13684), and anti-MLH1 (ES05, DAKO M3640) on Dako autostainers at Sapporo Clinical Laboratory. Both intraepithelial and stromal CD8^+^ cells in 10 high-power fields (HPF, ×400) were counted by pathologists in a blinded fashion. Cells inside and outside of the tumor parenchyma were histologically defined as intraepithelial and stromal cells, respectively.

### WES and mutation calling.

Genomic DNA was prepared using the Allprep DNA/RNA/Protein Kit (Qiagen) from CRC and matched normal tissues, or patient PBMCs. Exome capture libraries were prepared using SureSelect Human All Exon V6 (Agilent), and sequencing was performed using NovaSeq 6000 (Illumina) with 150 bp paired-end reads with a target depth of 150*×* coverage per sample. For the CRC009 samples, mutation calling was performed using the Genomon2 pipeline (https://genomon.readthedocs.io/ja/latest/) (61). For the CRC052, -054, -057, -059, and -111 samples, mutation calling was performed by Macrogen using DNA of CRC tissues and matched normal tissues. Briefly, the reads were mapped to the human reference genome (hg38) using BWA (v0.7.17). PCR duplicates were removed using Picard tools (2.18.2). Exome-based variants were called using GATK (v4.0.5.1). Detected variants were annotated using SnpEff (v4.3t) and filtered with dbSNP and SNPs from the 1000 Genomes Project (www.internationalgenome.org). Single nucleotide variants or indel mutations were called using the Mutect2 tool in the default parameter settings.

### RNA-seq.

Total RNA was isolated from CRC and matched normal tissues, or RAF9-reactive T cell clones, using Allprep DNA/RNA/Protein Kit (Qiagen) or TRIzol Reagent (Invitrogen) with validated quality of RNA integrity number (RIN) > 7. Poly A-selected libraries were prepared using the TruSeq Stranded mRNA LT Sample Prep Kit (Illumina). The libraries were sequenced on NovaSeq 6000 (Illumina) with 200M (cancer and normal tissues) or 40M (T cell clones) of 100 bp paired-end reads per sample. Read quality was validated and low-quality reads, adaptor sequences, contaminant DNA, or PCR duplicates were removed using FastQC (v0.11.7) and Trimmomatic (v0.38). The reads were mapped to the human reference genome (hg38) using HISAT2 (v2.1.0) and Bowtie2 (v2.3.4.1). The abundance of genes or transcripts was calculated as TPM using StringTie (v2.1.3b), and only genes with TPM > 0 were considered as expressed. Library preparation and sequencing were performed by Macrogen. The resulting data were analyzed using MCP-counter and MiXCR to calculate tissue infiltrating cell populations and TCR profiles, respectively (29, 43).

### Isolation of HLA class I ligands from tissues.

We followed the established protocol with slight modifications (62). Briefly, more than 1.5 g of each frozen tissues was ground under cryogenic conditions and lysed with lysate buffer containing 0.25% sodium deoxycholate (Wako), 0.2 mM iodoacetamide (Wako), 1 mM EDTA (Dojindo), protease inhibitor cocktail (P8340, MilliporeSigma), 1 mM PMSF (MilliporeSigma), and 1% octyl-β-D glucopyranoside (Dojindo) in DPBS (Thermo Fisher Scientific). The peptide–HLA-A24 complexes were captured using affinity chromatography of HLA-A24 mAb coupled to CNBr-activated Sepharose 4B (GE Healthcare) overnight. The HLA ligands were eluted with 0.2% TFA (Thermo Fisher Scientific) and desalted using Sep-Pak tC18 (Waters) with 28% ACN (Kanto Chemical) in 0.1% TFA and ZipTip U-C18 (MilliporeSigma) with 50% ACN in 1% FA (Wako). The samples were dried using vacuum centrifugation at 25°C and resuspended in 5% ACN in 0.1% TFA for liquid chromatography–MS/MS (LC-MS/MS) analysis. For the preparation of the HLA-A24 mAb, C7709A2 hybridoma (gifted by P.G. Coulie, Institut de Duve, Brussels, Belgium) was cultured in Hybridoma serum-free medium (SFM) (Thermo Fisher Scientific) supplemented with 1% penicillin/streptomycin in CELLine Bioreactor Flasks (CL1000, Corning). Condensed mAbs were collected and purified using HiTrap Protein G HP (GE Healthcare).

### LC-MS/MS.

Samples containing HLA-A24 ligands isolated from tissues were loaded into a nano-flow LC (Easy-nLC 1000 system, Thermo Fisher Scientific) online-coupled to an Orbitrap mass spectrometer equipped with a nanospray ion source (Q Exactive Plus, Thermo Fisher Scientific). Nano-flow LC separation was performed with a linear gradient ranging from 3% to 30% buffer B (100% ACN and 0.1% FA) at a flow rate of 300 nL/min for 80 minutes using a 75 μm × 20 cm capillary column with a particle size of 3 μm (NTCC-360, Nikkyo Technos). In MS, survey scan spectra were acquired at a resolution of 70,000 at 200 *m/z* with an AGC target value of 3e6 ions and a maximum IT of 100 ms, ranging from 350 to 2000 *m/z* with charge states between 1^+^ and 4^+^. A data-dependent top 10 method was employed. MS/MS resolution was 17,500 at 200 *m/z* with an AGC target value of 1e5 ions and a maximum IT of 120 ms.

### Proteogenomic identification of neoantigens and nonmutated HLA class I ligands.

MS/MS data were searched against personalized custom databases (see below) using the Sequest HT along with the Percolator algorithm on the Proteome Discoverer 2.3 platform (Thermo Fisher Scientific). The tolerance of precursor and fragment ions was set at 10 ppm and 0.02 Da, respectively. The oxidation of methionine (+15.995 Da) was selected as a dynamic modification. No specific enzyme was selected for the search. Concatenated target-decoy selection was validated based on *q* values, and a FDR of 0.01 was used in the Percolator node as a peptide detection threshold. A customized database for MS data search was constructed per tissue using Python scripts. Each database comprised 2 sets of sequences: (a) the GENCODE protein-coding transcript translation sequences (release 31) and (b) the protein sequences altered by the missense or frameshift mutation in the sample, starting from 30 amino acids upstream of a mutated residue and ending with 30 amino acid downstream residues (missense) or stop codons (frameshift). Only the protein sequences with source gene expression (TPM > 0) were registered in both data sets. Only the 8–11 mer peptides with %rank score (NetMHCpan-4.1) < 2.0 were counted as natural HLA-A*24:02 ligands for both mutated and nonmutated antigens.

### Synthetic peptides and peptide-HLA class I stabilization assay.

Synthetic peptides (RYLAVAAVF, RAF9; RYLTVAAVF, RTF9; RYLDWIKKI, RI9; IYQGRLWAF, IF9; EYVSQHLVF, EF9; KYLEKYYNL, KL9; KYLTAEAFGF, KF10; KYQPRVHVI, KI9; KYMYFTVVM, KM9; QYDPVAALF, CMV_pp65_; RYLRDQQLLGI, HIV_env584–594_; and GYISPYFINTSK, GK12) with > 80% purity were purchased from MilliporeSigma (CMV_pp65_, HIV_env584–594_, and GK12) and Cosmo Bio (RAF9, RTF9, RI9, IF9, EF9, KL9, KF10, KI9, and KM9). The stability of peptide–HLA-class I complexes on cell surfaces was assessed using T2-A24 cells. The TAP-deficient T2-A24 cells were precultured overnight at 25°C. The following day, the cells were incubated with the indicated peptides in a range of the indicated concentrations for 1 hour at 25°C, followed by incubation for 3 hours at 37°C. Next, the cells were stained with the HLA-A24 mAbs (C7709A2), followed by goat anti–mouse IgG-FITC (KPL, 02-18-09), and analyzed using FACS Canto II (BD Biosciences). The stability was calculated by the difference in mean fluorescence intensity values (ΔMFI) between samples with and without a peptide pulse.

### Establishment and characterization of neoantigen-reactive TIL clones.

Bulk TILs were cultured in the presence of 1 μM RAF9 peptide, and CD8^+^ and 4-1BB^+^ (CD137^+^) cell populations were isolated using FACS Aria II (BD Biosciences) and sorted into U-bottom 96-well plates. Each single cell was expanded in complete AIM-V medium supplemented with 3000 U/mL IL-2, 5 μg/mL phytohemagglutinin (PHA, WAKO), and irradiated human PBMCs. For T cell phenotype analysis, the cells were prestained with human FcR blocking reagent (Clear Back, MBL, MTG-001) and stained with following antibodies at 4°C for 20 minutes: anti–CD3 PE (UCHT1, BioLegend, 300408), anti–CD8 PE (SK1, BD Biosciences, 340046), anti–CD28 PE (CD28.2, BioLegend, 302907), anti–CD45RA FITC (HI100, BD Biosciences, 555488), anti–CD45RO PE (UCHL1, BioLegend, 304206), anti–CD62L PE-Cy5 (DREG-56, BD Biosciences, 555545), anti–CCR7 PE (150503, R&D Systems, FAB197P), anti–PD-1 APC (EH12.2H7, BioLegend, 329908), and anti–CD39 PerCP/Cy5.5 (A1, BioLegend, 328218).

### T cell analysis using flow cytometry.

The functions and phenotypes of TILs or CD8^+^ TIL clones were analyzed using FACS Canto II (BD Biosciences) with FACS Diva (BD Biosciences) or FlowJo (Tree Star Inc.). For T cell functions, CD107a mobilization and intracellular IFN-γ production were assessed using Fixation/Permeabilization Solution Kit with BD GolgiPlug (BD Biosciences), according to the manufacturer’s instructions. Briefly, T2-A24 cells were pulsed with 1 μM of the indicated synthetic peptides for 1 hour at 25°C. Expanded CRC111 TILs were cultured with the peptide-pulsed T2-A24 cells for 4 hour at 37°C in the presence of human FcR blocking reagent (Clear Back, MBL MTG-001), GolgiPlug, and anti–CD107a PE (H4A3, BioLegend, 328608). Next, the cells were stained with anti–CD3 PE-Cy7 (SK7, BD Biosciences, 341091) and anti–CD8 PC5 (T8, Beckman Coulter, 6607011), followed by permeabilization and staining with anti–IFN-γ FITC (B27, BD Biosciences, 552887). Likewise, 4-1BB was used as an activation marker. TILs were cultured with T2-A24 cells pulsed with or without 1 μM of indicated synthetic peptides for 24 hours at 37°C and stained with anti–CD3 PE-Cy7 (SK7, BD Biosciences, 341091), anti–CD8 PE (SK1, BD Biosciences, 340046), and anti–4-1BB FITC (REA765, Miltenyi Biotec, 130-110-765). The other antibodies used for cell staining are as follows: anti–CD3 PE (UCHT1, BioLegend, 300408), anti–CD8 FITC (T8, Beckman Coulter, 6603861), anti–PD-1 APC (EH12.2H7, BioLegend, 329908), anti–CD45RO BV421 (UCHL1, BD Horizon, 562649), anti–CCR7 PerCP-Cy5.5 (150503, BD Pharmingen, 561144), anti–CD45RA PE-Cy7 (L48, BD Pharmingen, 337167), and anti–CD62L APC (DREG-56, BD Pharmingen, 561916). The HLA-A24 tetramers complexed with RAF9 or HIV_env584–592_ were purchased from MBL.

### IFN-γ ELISpot.

Antigen presenting cells (T2 or T2-A24) were incubated with or without 100 nM, or a range of indicated concentrations, of synthetic peptides for 2 hours at 25°C. Neoantigen-reactive CD8^+^ T cell clones were mixed with the antigen-presenting cells at a 1:1 ratio and were cultured on the human IFN-γ ELISpot plate (BD Biosciences) for 24 hours at 37°C, according to the manufacturer’s instructions. The contents of the plate were reacted with a biotinylated anti–human IFN-γ for 2 hours at 25°C, followed by the ELISpot Streptavidin-HRP for 1 hour at 25°C, and positive spots were visualized using the ELISpot AEC Substrate Set (BD Biosciences). In a peptide titration for measuring functional avidity, the peptide concentration of a half-maximal IFN-γ production was calculated as EC_50_.

### LDH cytotoxicity assay.

T2-A24 cells preincubated with or without 20 μM synthetic peptides for 2 hours at 25°C served as target cells. Neoantigen-reactive T cell clones were cultured with 1 × 10^4^ target cells at indicated effector/target ratios (E/T) for 6 hours at 37°C. The amount of LDH released from lysed target cells was measured using the LDH Cytotoxicity Detection Kit (Takara Bio) according to the manufacturer’s instructions. The percentage of LDH released was calculated as follows:

(Equation 1)
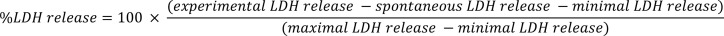


Spontaneous, minimal, and maximal LDH releases were values of T cell alone, target cells alone, and target cells treated with 2% NP-40 (Sigma-Aldrich), respectively.

### Isolation of TCRαβ from a TIL clone.

TCRαβ sequences were isolated from 1E8, an RAF9-reactive clone, via 5′-rapid amplification of cDNA ends (RACE) PCR using a SMARTer RACE 5′/3′ kit (Takara Bio) as previously described (63). The following primers were used for amplification: a supplied 5′-RACE primer (first PCR for both TCRα and -β), 5′-GGAGAGTTCCCTCTGTTTGGAGAG-3′(first PCR for TCRα), 5′-TTTATCGTCGACCACTGTGCTGGCGGCCGCTCGAGTCAGAAATCCTTTCTCTTGACC-3′ and 5′-ATCGTCGACCACTGTGCTGGCGGCCGCTCGAGCTAGCCTCTGGAATCCTTTCTCTTGACC-3′(first and second PCR for TCRβ), 5′-GTGTGGTGGTACGGGAATTCAAGCAGTGGTATCAACGCAGAGT-3′ (supplied 5′-RACE primer with modified extension, second PCR for both TCRα and β), and 5′-ACCACTGTGCTGGCGGCCGCTCAGCTGGACCACAGCCGCAGCG-3′ (second PCR for TCRα). The second PCR products of TCRα and -β were cloned into a pMX retroviral vector using the NEBuilder HiFi DNA Assembly Master Mix (NEB) according to the manufacturer’s instructions. The PCR products were sequenced, and their TCR clonotypes were determined according to the IMGT database (http://www.imgt.org).

### TCR transduction.

In the following experiments, PLAT-A cells (Cosmo Bio, RV-102) were transfected with the corresponding pMX vectors using TransIT293 (Mirus Bio), and the culture supernatant was subsequently used for transduction into a packaging cell line, PG13 (ATCC, CRL-10686). The culture supernatant of the infected PG13 cells was used for transduction into SUP-T1 cells (ATCC, CRL-1942) or HD PBMCs. For transduction into SUP-T1 cells, pMX vectors for RAF9-TCRα and -β sequences were simultaneously introduced. For transduction to HD PBMCs, TCRβ and TCRα were concatenated and cloned into a single pMX vector, as previously described (64). Briefly, the 3′ region of TCRβ was followed by a furin cleavage site, an SGSG spacer sequence, a P2A sequence (5′- AGAGCCAAGAGATCTGGCAGCGGCGCCACAAACTTCAGCCTGCTGAAACAGGCTGGCGACGTGGAAGAGAACCCCGGACCT-3′), and the 5′ region of TCRα. PBMCs were isolated from HD peripheral blood using Lymphoprep (Cosmo Bio) and transduced using the collected PG13 supernatant after stimulation with 50 ng/mL anti-CD3 mAb (OKT3, BioLegend, 317302) for 3 days. The transduced cells were used for flow cytometry or the IFN-γ ELISpot assay, as described above. The target cells for the ELISpot assay were T2-A24 cells pulsed with 100 nM of peptides or 293T-A24 cells transiently transfected with RAF9 minigene constructs. The RAF9-specific CD8^+^ fraction of the transduced PBMCs (HD1) was also enriched using a cell sorter (FACS Aria II; BD Biosciences) with RAF9–HLA-A24 tetramer PE and anti–CD8 FITC (T8, Beckman Coulter 6603861). More than 98% of the enriched cells were double-positive for the tetramer and CD8. The enriched cells were then expanded in complete AIM-V medium supplemented with 100 U/mL IL-2, 30 ng/mL anti-CD3 (OKT3, BioLegend, 317302) and irradiated PBMCs for 2 weeks, and they were used for the LDH assay as described above.

### DNA constructs for antigen expression.

Sequences encoding RAF9 or RTF9 antigens following a signal sequence (RAF9: 5′-CGATACCTCGCCGTGGCTGCTGTCTTC-3′, RTF9: 5′-CGATACCTCACCGTGGCTGCTGTCTTC-3′) were synthesized and cloned into pcDNA3.1. For the IFN-γ ELISpot assay, 293T-A24 cells were transfected with the corresponding vectors, and they served as targets 48 hours after transfection.

### Data availability.

MS raw data and personalized FASTA files generated based on WES and RNA-seq data have been deposited to the ProteomeXchange Consortium via the jPOSTrepo partner repository (https://repository.jpostdb.org) with the data set identifier PXD026573.

### Statistics.

Data analysis was performed using GraphPad Prism (v8). Data are shown as the mean ± SEM.

### Study approval.

The study was conducted with the approval of the IRB (no. 312-1134) and the Research Ethics Committee of Sapporo Medical University (no. 29-2-69).

## Author contributions

TH, IT, and FH prepared and provided the patient samples. ST and K. Murata performed the experiments. MN helped develop the proteogenomic pipeline. YN and SO performed the WES. K. Matsuo performed IHC. HI performed HLA genotyping. TH, ST, YH, SH, NS, and TK interpreted the experiments. TK conceived and designed the project. ST and TK prepared and wrote the manuscript. TT supervised the project. All authors reviewed and approved the contents of the manuscript.

## Supplementary Material

Supplemental data

Supplemental table 1

Supplemental table 2

Supplemental table 3

## Figures and Tables

**Table 1:**
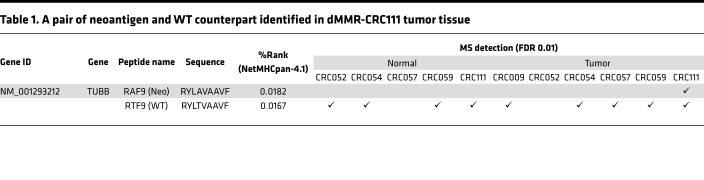
A pair of neoantigen and WT counterpart identified in dMMR-CRC111 tumor tissue

**Table 2:**
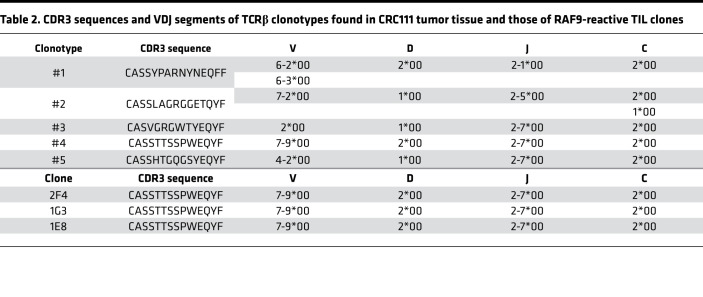
CDR3 sequences and VDJ segments of TCRβ clonotypes found in CRC111 tumor tissue and those of RAF9-reactive TIL clones

## Version 1 06/29/2021

In-Press Preview

## Version 2 07/22/2021

Electronic publication

## References

[1] Boland CR (1998). A National Cancer Institute workshop on microsatellite instability for cancer detection and familial predisposition: development of international criteria for the determination of microsatellite instability in colorectal cancer. Cancer Res.

[2] Jiricny J (2006). The multifaceted mismatch-repair system. Nat Rev Mol Cell Biol.

[3] Le DT (2015). PD-1 Blockade in tumors with mismatch-repair deficiency. N Engl J Med.

[4] Le DT (2017). Mismatch-repair deficiency predicts response of solid tumors to PD-1 blockade. Science.

[5] Yarchoan M (2017). Tumor mutational burden and response rate to PD-1 inhibition. N Engl J Med.

[6] Schumacher TN, Schreiber RD (2015). Neoantigens in cancer immunotherapy. Science.

[7] Gubin MM (2014). Checkpoint blockade cancer immunotherapy targets tumour-specific mutant antigens. Nature.

[8] Germano G (2017). Inactivation of DNA repair triggers neoantigen generation and impairs tumour growth. Nature.

[9] Tran E (2014). Cancer immunotherapy based on mutation-specific CD4+ T cells in a patient with epithelial cancer. Science.

[10] Tran E (2016). T-cell transfer therapy targeting mutant KRAS in cancer. N Engl J Med.

[11] Zacharakis N (2018). Immune recognition of somatic mutations leading to complete durable regression in metastatic breast cancer. Nat Med.

[12] Carreno BM (2015). Cancer immunotherapy. A dendritic cell vaccine increases the breadth and diversity of melanoma neoantigen-specific T cells. Science.

[13] Ott PA (2017). An immunogenic personal neoantigen vaccine for patients with melanoma. Nature.

[14] Sahin U (2017). Personalized RNA mutanome vaccines mobilize poly-specific therapeutic immunity against cancer. Nature.

[15] Vitiello A, Zanetti M (2017). Neoantigen prediction and the need for validation. Nat Biotechnol.

[16] Nesvizhskii AI (2014). Proteogenomics: concepts, applications and computational strategies. Nat Methods.

[17] Zhang B (2019). Clinical potential of mass spectrometry-based proteogenomics. Nat Rev Clin Oncol.

[18] Yadav M (2014). Predicting immunogenic tumour mutations by combining mass spectrometry and exome sequencing. Nature.

[19] Ebrahimi-Nik H (2019). Mass spectrometry driven exploration reveals nuances of neoepitope-driven tumor rejection. JCI Insight.

[20] Kalaora S (2016). Use of HLA peptidomics and whole exome sequencing to identify human immunogenic neo-antigens. Oncotarget.

[21] Kochin V (2017). HLA-A24 ligandome analysis of colon and lung cancer cells identifies a novel cancer-testis antigen and a neoantigen that elicits specific and strong CTL responses. Oncoimmunology.

[22] Newey A (2019). Immunopeptidomics of colorectal cancer organoids reveals a sparse HLA class I neoantigen landscape and no increase in neoantigens with interferon or MEK-inhibitor treatment. J Immunother Cancer.

[23] Bassani-Sternberg M (2016). Direct identification of clinically relevant neoepitopes presented on native human melanoma tissue by mass spectrometry. Nat Commun.

[24] Kalaora S (2018). Combined analysis of antigen presentation and T-cell recognition reveals restricted immune responses in Melanoma. Cancer Discov.

[25] Dolcetti R (1999). High prevalence of activated intraepithelial cytotoxic T lymphocytes and increased neoplastic cell apoptosis in colorectal carcinomas with microsatellite instability. Am J Pathol.

[26] Hegde PS (2016). The where, the when, and the how of immune monitoring for cancer immunotherapies in the era of checkpoint inhibition. Clin Cancer Res.

[27] Chen DS, Mellman I (2017). Elements of cancer immunity and the cancer-immune set point. Nature.

[28] Sun C (2018). Regulation and function of the PD-L1 checkpoint. Immunity.

[29] Becht E (2016). Estimating the population abundance of tissue-infiltrating immune and stromal cell populations using gene expression. Genome Biol.

[30] Bassani-Sternberg M (2015). Mass spectrometry of human leukocyte antigen class I peptidomes reveals strong effects of protein abundance and turnover on antigen presentation. Mol Cell Proteomics.

[31] Abelin JG (2017). Mass spectrometry profiling of HLA-associated peptidomes in mono-allelic cells enables more accurate epitope prediction. Immunity.

[32] Shraibman B (2019). Identification of tumor antigens among the HLA peptidomes of glioblastoma tumors and plasma. Mol Cell Proteomics.

[33] Sarkizova S (2020). A large peptidome dataset improves HLA class I epitope prediction across most of the human population. Nat Biotechnol.

[34] Stopfer LE (2020). Multiplexed relative and absolute quantitative immunopeptidomics reveals MHC I repertoire alterations induced by CDK4/6 inhibition. Nat Commun.

[35] Maby P (2015). Correlation between density of CD8+ T-cell infiltrate in microsatellite unstable colorectal cancers and frameshift mutations: a rationale for personalized immunotherapy. Cancer Res.

[36] Tran E (2015). Immunogenicity of somatic mutations in human gastrointestinal cancers. Science.

[37] Parkhurst MR (2019). Unique neoantigens arise from somatic mutations in patients with gastrointestinal cancers. Cancer Discov.

[38] Bobisse S (2018). Sensitive and frequent identification of high avidity neo-epitope specific CD8 ^+^ T cells in immunotherapy-naive ovarian cancer. Nat Commun.

[39] Galon J (2006). Type, density, and location of immune cells within human colorectal tumors predict clinical outcome. Science.

[40] Gros A (2014). PD-1 identifies the patient-specific CD8^+^ tumor-reactive repertoire infiltrating human tumors. J Clin Invest.

[41] Simoni Y (2018). Bystander CD8^+^ T cells are abundant and phenotypically distinct in human tumour infiltrates. Nature.

[42] Tumeh PC (2014). PD-1 blockade induces responses by inhibiting adaptive immune resistance. Nature.

[43] Bolotin DA (2015). MiXCR: software for comprehensive adaptive immunity profiling. Nat Methods.

[44] Robins HS (2009). Comprehensive assessment of T-cell receptor beta-chain diversity in alphabeta T cells. Blood.

[45] Gong Q (2017). Assessment of T-cell receptor repertoire and clonal expansion in peripheral T-cell lymphoma using RNA-seq data. Sci Rep.

[46] Hinrichs CS (2009). Adoptively transferred effector cells derived from naive rather than central memory CD8+ T cells mediate superior antitumor immunity. Proc Natl Acad Sci U S A.

[47] Hinrichs CS (2011). Human effector CD8+ T cells derived from naive rather than memory subsets possess superior traits for adoptive immunotherapy. Blood.

[48] Neefjes J (2011). Towards a systems understanding of MHC class I and MHC class II antigen presentation. Nat Rev Immunol.

[49] Shionoya Y (2017). Loss of tapasin in human lung and colon cancer cells and escape from tumor-associated antigen-specific CTL recognition. Oncoimmunology.

[50] Durgeau A (2018). Human preprocalcitonin self-antigen generates TAP-dependent and -independent epitopes triggering optimised T-cell responses toward immune-escaped tumours. Nat Commun.

[51] Marijt KA, van Hall T To TAP or not to TAP: alternative peptides for immunotherapy of cancer. Curr Opin Immunol.

[52] Schreiber RD (2011). Cancer immunoediting: integrating immunity’s roles in cancer suppression and promotion. Science.

[53] Angelova M (2018). Evolution of metastases in space and time under immune selection. Cell.

[54] Zaretsky JM (2016). Mutations associated with acquired resistance to PD-1 blockade in Melanoma. N Engl J Med.

[55] Marty R (2017). MHC-I genotype restricts the oncogenic mutational landscape. Cell.

[56] Rosenthal R (2019). Neoantigen-directed immune escape in lung cancer evolution. Nature.

[57] Chalabi M (2020). Neoadjuvant immunotherapy leads to pathological responses in MMR-proficient and MMR-deficient early-stage colon cancers. Nat Med.

[58] Laumont CM (2018). Noncoding regions are the main source of targetable tumor-specific antigens. Sci Transl Med.

[59] Chong C (2020). Integrated proteogenomic deep sequencing and analytics accurately identify non-canonical peptides in tumor immunopeptidomes. Nat Commun.

[60] Dudley ME (2003). Generation of tumor-infiltrating lymphocyte cultures for use in adoptive transfer therapy for melanoma patients. J Immunother.

[61] Yokoyama A (2019). Age-related remodelling of oesophageal epithelia by mutated cancer drivers. Nature.

[62] Kowalewski DJ, Stevanovic S (2013). Biochemical large-scale identification of MHC class I ligands. Methods Mol Biol.

[63] Ochi T (2015). Optimization of T-cell reactivity by exploiting TCR chain centricity for the purpose of safe and effective antitumor TCR gene therapy. Cancer Immunol Res.

[64] Yang S (2008). Development of optimal bicistronic lentiviral vectors facilitates high-level TCR gene expression and robust tumor cell recognition. Gene Ther.

