# Supplementary material for: Proteogenomic identification of an immunogenic HLA class I neoantigen in mismatch repair–deficient colorectal cancer tissue
Source: JCI Insight. 2021 Jul 22;6(14):e146356. doi: 10.1172/jci.insight.146356 (PMC8410045; doi:10.1172/jci.insight.146356)
Supplement: Supplemental data [file jciinsight-6-146356-s233.pdf]

Supplemental Information

Figure S1: Immunohistochemistry (IHC) of CRC tissues

Formalin-fixed paraffin embedded (FFPE) tissues were mounted and stained with hematoxylin and eosin (HE) or with following antibodies on DAKO autostainers: anti-MLH1 (ES05, DAKO), anti-pan HLA class I (EMR8-5, Hokudo), anti-CD8 (C8/114B, DAKO), and anti-PD-L1 (E1L3N, CellSignaling). Magnification, ×200. CRC111 is a mismatch repair deficient (dMMR) tumor accompanied by loss of MLH1 expression. All the other CRCs are mismatch repair proficient (pMMR) tumors, which express both MSH6 and PMS2 in addition to MLH1 (data not shown). Primary sites, histological tumor types, and stages of six CRC tissues are shown.

Figure S2: Proteogenomic pipeline for direct neoantigen identification

CRC tumor tissues or patient-matched normal mucosa were analyzed. A personalized database for MS search was constructed per sample (upper panel). Each database contained altered amino-acid sequences determined by WES as well as reference protein sequences. Only the protein sequences with source gene expression (TPM > 0) determined by RNA-seq were enrolled. Meanwhile, HLA-A24 ligands were biochemically captured and analyzed using LC-MS/MS (lower panels). The obtained MS/MS signals were searched against the sample-specific database, yielding a comprehensive list of non-mutated HLA-A24 ligands and mutation-derived neoantigens, both of which were naturally processed and presented by the sample.

Figure S3: Expression of the *TUBB* gene across tissue and organ types

Abundant gene expression of *TUBB*, which encodes the RAF9 neoantigen in CRC111, across tissue and organ types (GTEx: https://www.gtexportal.org/home/).

Figure S4: HLA class I restriction of a T-cell response to RAF9

IFNγ ELISpot assay of an RAF9-reactive $CD8^+$ T cell clone (2F4) in response to T2-A24 pulsed with 100 nM RAF9 peptide. The responses were assessed in the presence of 100 μg/mL of MHC antibodies. These results validated the HLA-A24 restriction of the T cell response to RAF9. Data represent means with SEM (n=3), and p-values were calculated using a two-tailed t-test (** $p < 0.01$).

Figure S5: Enrichment of the RAF9-specific fraction of TCR-transduced PBMCs

Flow cytometry of HD1-derived PBMCs transduced with a concatenated RAF9-reactive TCRα and β sequences. The fraction positive for the RAF9-HLA-A24 tetramer and CD8 (2.41% of $CD8^+$ cells) was enriched using a cell sorter (FACS Aria II, BD). Approximately 99.9% of the sorted cells were positive for the tetramer and CD8.

Table S1: HLA class I genotypes of patients with CRC

Table S2: List of non-mutated HLA-A24 ligands identified in CRC tissues and matched normal tissues

Table S3: List of TAA candidates used for dMMR-CRC111 TIL analysis

Figure S1

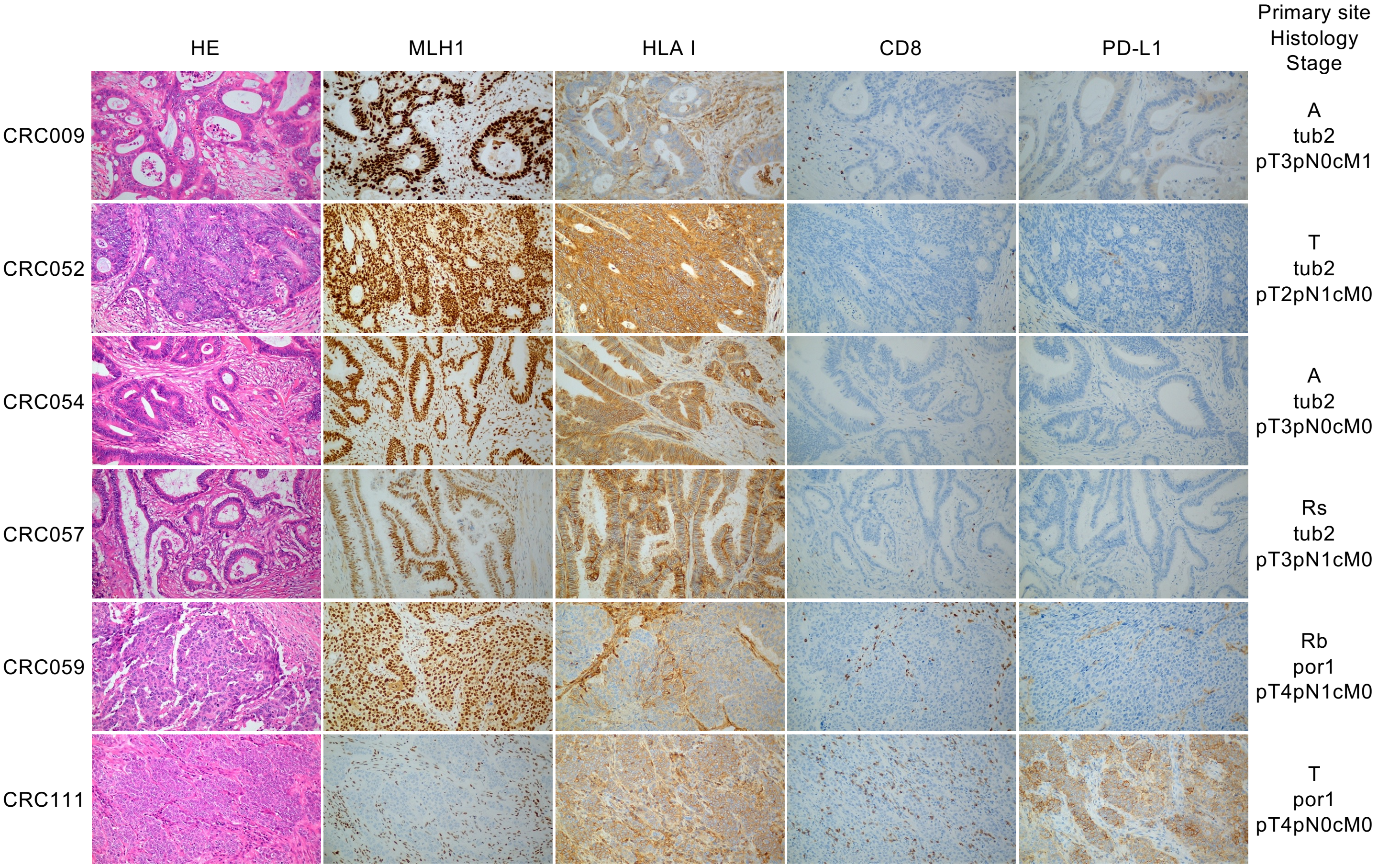
HE
MLH1
HLA I
CD8
PD-L1
Primary site
Histology
Stage
CRC009
A
tub2
pT3pN0cM1
CRC052
T
tub2
pT2pN1cM0
CRC054
A
tub2
pT3pN0cM0
CRC057
Rs
tub2
pT3pN1cM0
CRC059
Rb
por1
pT4pN1cM0
CRC111
T
por1
pT4pN0cM0

Figure S2

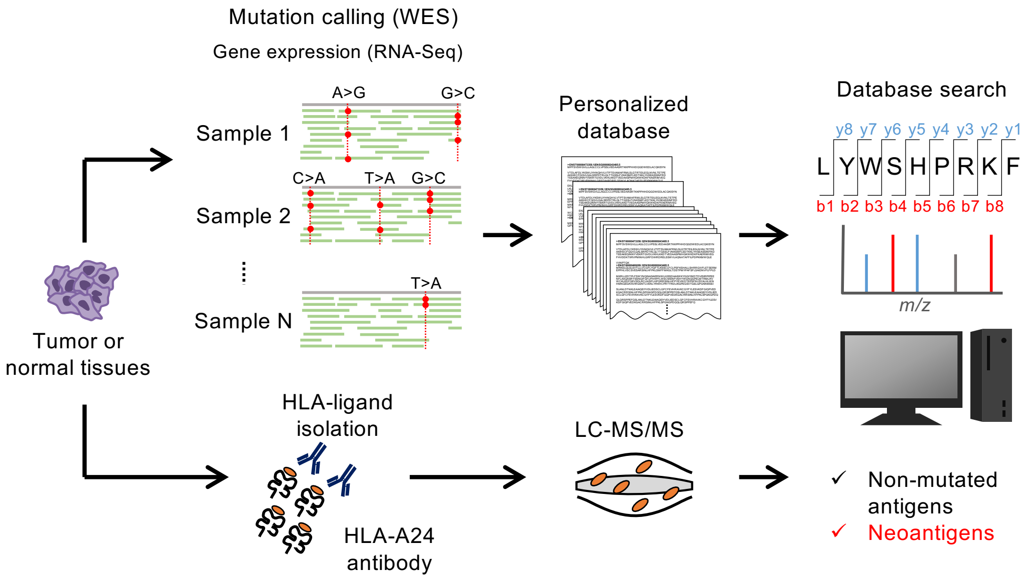
Mutation calling (WES)
Gene expression (RNA-Seq)
Sample 1
A>G
G>C
Sample 2
C>A
T>A
G>C
Sample N
T>A
Tumor or normal tissues
Personalized database
Database search
y8 y7 y6 y5 y4 y3 y2 y1
L Y W S H P R K F
b1 b2 b3 b4 b5 b6 b7 b8
m/z
HLA-ligand isolation
HLA-A24 antibody
LC-MS/MS
✓ Non-mutated antigens
✓ Neoantigens

Figure S3

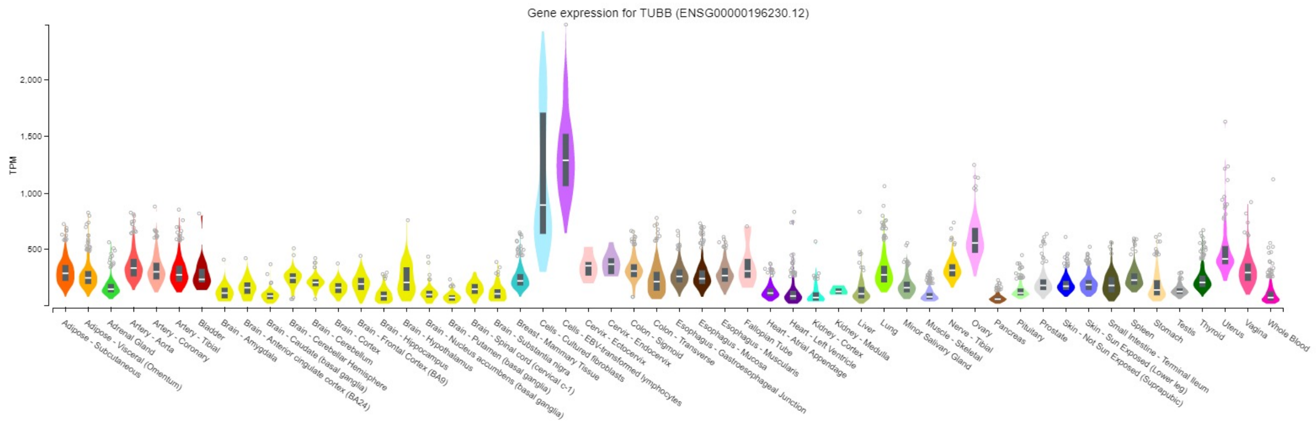
Gene expression for TUBB (ENSG00000196230.12)
TPM
2,000
1,500
1,000
500
Adipose - Subcutaneous
Adipose - Visceral (Omentum)
Adrenal Gland
Artery - Aorta
Artery - Coronary
Artery - Tibial
Bladder
Brain - Amygdala
Brain - Anterior cingulate cortex (BA24)
Brain - Caudate (basal ganglia)
Brain - Cerebellar Hemisphere
Brain - Cerebellum
Brain - Cortex
Brain - Frontal Cortex (BA9)
Brain - Hippocampus
Brain - Hypothalamus
Brain - Nucleus accumbens (basal ganglia)
Brain - Putamen (basal ganglia)
Brain - Spinal cord (cervical c-1)
Brain - Substantia nigra
Breast - Mammary Tissue
Cells - Cultured fibroblasts
Cells - EBV-transformed lymphocytes
Cervix - Ectocervix
Cervix - Endocervix
Colon - Sigmoid
Colon - Transverse
Esophagus - Gastroesophageal Junction
Esophagus - Mucosa
Esophagus - Muscularis
Fallopian Tube
Heart - Atrial Appendage
Heart - Left Ventricle
Kidney - Cortex
Kidney - Medulla
Liver
Lung
Minor Salivary Gland
Muscle - Skeletal
Nerve - Tibial
Ovary
Pancreas
Pituitary
Prostate
Skin - Not Sun Exposed (Suprapubic)
Skin - Sun Exposed (Lower leg)
Small Intestine - Terminal Ileum
Spleen
Stomach
Testis
Thyroid
Uterus
Vagina
Whole Blood

Figure S4

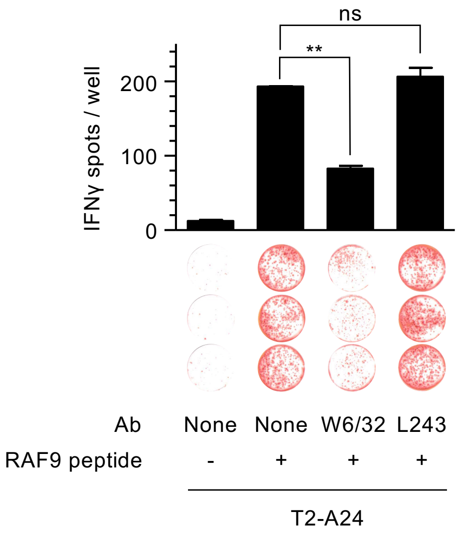

ns
**
IFNγ spots / well
200
100
0
Ab
None
None
W6/32
L243
RAF9 peptide
-
+
+
+
T2-A24

Figure S5

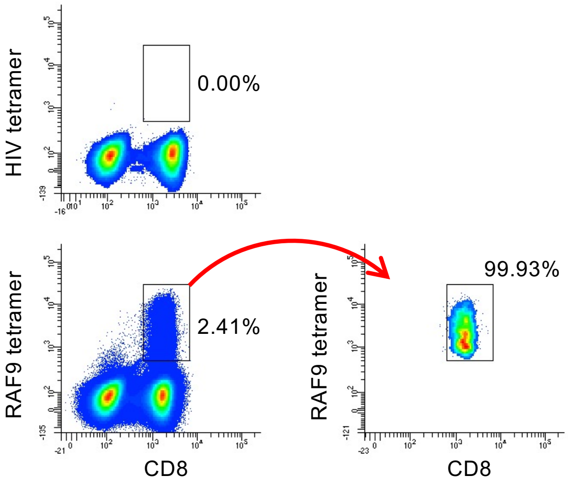
HIV tetramer
0.00%
RAF9 tetramer
2.41%
CD8
RAF9 tetramer
99.93%
CD8
